# Spatial heterogeneity response of soil salinization inversion cotton field expansion based on deep learning

**DOI:** 10.3389/fpls.2024.1437390

**Published:** 2024-11-12

**Authors:** Jinming Zhang, Jianli Ding, Jinjie Wang, Zihan Zhang, Jiao Tan, Xiangyu Ge

**Affiliations:** ^1^ College of Geography and Remote Sensing Sciences, Xinjiang University, Urumqi, China; ^2^ Xinjiang Key Laboratory of Oasis Ecology, Xinjiang University, Urumqi, China; ^3^ Xinjiang Institute of Technology, Aksu, China

**Keywords:** deep learning, soil salinization, cotton field expansion, MGWR model, spatial heterogeneity

## Abstract

Soil salinization represents a significant challenge to the ecological environment in arid areas, and digital mapping of soil salinization as well as exploration of its spatial heterogeneity with crop growth have important implications for national food security and salinization management. However, the machine learning models currently used are deficient in mining local information on salinity and do not explore the spatial heterogeneity of salinity impacts on crops. This study developed soil salinization inversion models using CNN (Convolutional Neural Network), LSTM (Long Short-Term Memory Network), and RF (Random Forest) models based on 97 field samples and feature variables extracted from Landsat-8 imagery. By evaluating the accuracy, the best-performing model was selected to map soil salinity at a 30m resolution for the years 2013 and 2022, and to explore the relationship between soil electrical conductivity (EC) values and the expansion of cotton fields as well as their spatial correlation. The results indicate that:(1) The CNN performs best in prediction, with an R^2^ of 0.84 for the training set and 0.73 for the test set, capable of capturing more local salinity information. (2) The expansion of cotton fields has reduced the level of soil salinization, with the area of severely salinized and saline soils in newly added cotton fields decreasing from 177.91 km^2^ and 381.46 km^2^ to 19.49 km^2^ and 1.12 km^2^, respectively. (3) Regions with long-term cotton cultivation and newly reclaimed cotton fields exhibit high sensitivity and vulnerability to soil salinity. This study explores the excellent performance of deep learning in salinity mapping and visualizes the spatial distribution of cotton fields that are highly sensitive to soil salinity, providing a scientific theoretical basis for accurate salinity management.

## 1 Introduction

Soil salinization poses a significant challenge to global agriculture, with saline soils now present in over 100 countries and regions worldwide (Abbas et al., 2013). For example, approximately one-quarter of the irrigated land in Pakistan has been salinized, with annual economic costs estimated between $0.26 and $9.4 billion. The yields of wheat and rice grown in saline-alkali soils have decreased by 32% and 48%, respectively (Sheikh et al., 2022). Similarly, in Iraq's Mesopotamian region, crop yields in salinized farmland have decreased by 30-60% (Wu et al., 2018). In China, approximately 37.72% of the total irrigated land in Xinjiang is affected by salinization (Muhetaer et al., 2022).The accumulation of salt ions in the soil leads to physiological drought in plants, inhibits nutrient absorption, ultimately resulting in poor development, decreased yields, and even death (Ma and Tashpolat, 2023). Soil salinization results in a disruption of the water-salt balance in affected areas, which presents a significant threat to the ecological environment and biosphere (Zhang et al., 2022). In order to formulate optimized soil improvement policies to address the persistent degradation of land in typical arid oasis areas, the reversal of soil salinization has emerged as a significant area of investigation within the field of salinization.

Oases are a distinctive geographical phenomenon that emerge under specific natural geographical and climatic conditions. They represent major areas for human habitation and development (Huang et al., 2007). The Wei-Ku Oasis is one of the traditional agricultural areas in Xinjiang. Since the 1950s, the Chinese government has implemented policies to promote agricultural growth, which has led to a notable expansion of cultivated land (Shabiti et al., 2008). Based on long-term field surveys, our research team has found that in the past decade (2013-2022), humans have continuously reclaimed peripheral wasteland. Saline-alkali land has been improved into arable land through drainage, flushing, salt drainage, and appropriate agricultural measures, with cotton as the main crop type. Cotton, as a significant cash crop, has made outstanding contributions to the local economy. In previous studies, research on cotton has mainly focused on identifying planting distribution and predicting growth and yield. Exploring the relationship between cotton and soil salinization can contribute to the improvement of saline-alkali land, the safeguarding of China's red line of 120 million hectares of arable land and the assurance of food security are of paramount importance (Chen et al., 2019).

Over the past decade, there has been a significant advancement in the field of research pertaining to the reversal of soil salinization. The three elements of available remote sensing image data—spatial resolution, temporal resolution, and spectral resolution—have all improved, and inversion methods have evolved from traditional geostatistical analysis to the use of machine learning models. Geostatistical methods permit the analysis of the spatial distribution characteristics of soil salinity, employing semi-variance functions and Kriging interpolation (Gong et al., 2012; Rongjiang and Jingsong, 2009). Nevertheless, these methods do not exhibit significant variability. With the widespread use of machine learning algorithm since the late 20th century. It requires fewer parameters defined by researchers in soil salinity prediction studies, have higher computational speed and efficiency, and can handle advantages such as numerical ordinal and discrete predictor variables (Wang et al., 2019). The model effectively addresses the nonlinearity between soil and environmental factors. Commonly used machine learning models include RF (Ma et al., 2021), GBDT (Chen et al., 2021) and PLSR (Chengzhi et al., 2022). Over the past decade, the computer field has experienced a period of rapid development, deep learning has gradually emerged. It can be used not only for image recognition tasks but also for sequence prediction and other tasks. Representative deep learning models include CNN (Amarasinghe et al., 2017), LSTM (Hochreiter and Schmidhuber, 1997), BPNN (Hao et al., 2021), etc. By comparing the mapping results of deep learning models and machine learning models, it is possible to provide more robust scientific evidence for the regional management of soil salinization.

Currently, the main methods for exploring the relationship between two factors include Pearson correlation analysis (Gogtay and Thatte, 2017) and grey relational analysis (Chakraborty et al., 2023). However, these methods overlook their spatial heterogeneity. Geographical Detector (Yu et al., 2021) addresses this issue by better exploring the spatial heterogeneity between variables and explaining their interactions. However, the Geographical Detector cannot explore the local spatial expression of variables according to their correlations. The Multiscale Geographically Weighted Regression (MGWR) model (Fotheringham et al., 2017), as a local modelling method derived from the GWR model has been employed to analyze spatial relationships in ecological processes. Unlike the GWR model, it can search for the optimal bandwidth (scale) for regression analysis, thereby providing more detailed regression coefficient estimates, which allows for a more accurate capture of spatial heterogeneity. Consequently, this study introduces the MGWR model in an innovative manner to investigate the interrelationship between salinization and cotton fields, thereby providing scientific evidence for the improvement of salinization and the rational expansion of cotton fields. The proposed method will achieve local sustainable development.

In soil salinity prediction, previous studies primarily used machine learning models for regression predictions. Although good prediction results were achieved, they did not integrate the rapidly developing deep learning technologies of recent years. This study explores two established deep learning models, CNN and LSTM, as well as the most widely used machine learning model, RF. The optimal model is selected to create digital maps of soil salinity. Through the MGWR model, this study innovatively investigates the local expression of how soil salinity affects the spatial heterogeneity of cotton field yields, providing new directions for targeted and regional management of cotton field soil salinization. The main research objectives are as follows: (1) To compare the differences in the inversion effects and accuracy of soil salinization between the CNN, LSTM, and RF models to better depict soil salinity maps; (2) To map the distribution of cotton planting and predict yield distribution; (3) To study the improvement effects and spatial heterogeneity of cotton field expansion on saline-alkali land.

## 2 Materials and methods

**Figure 1:**
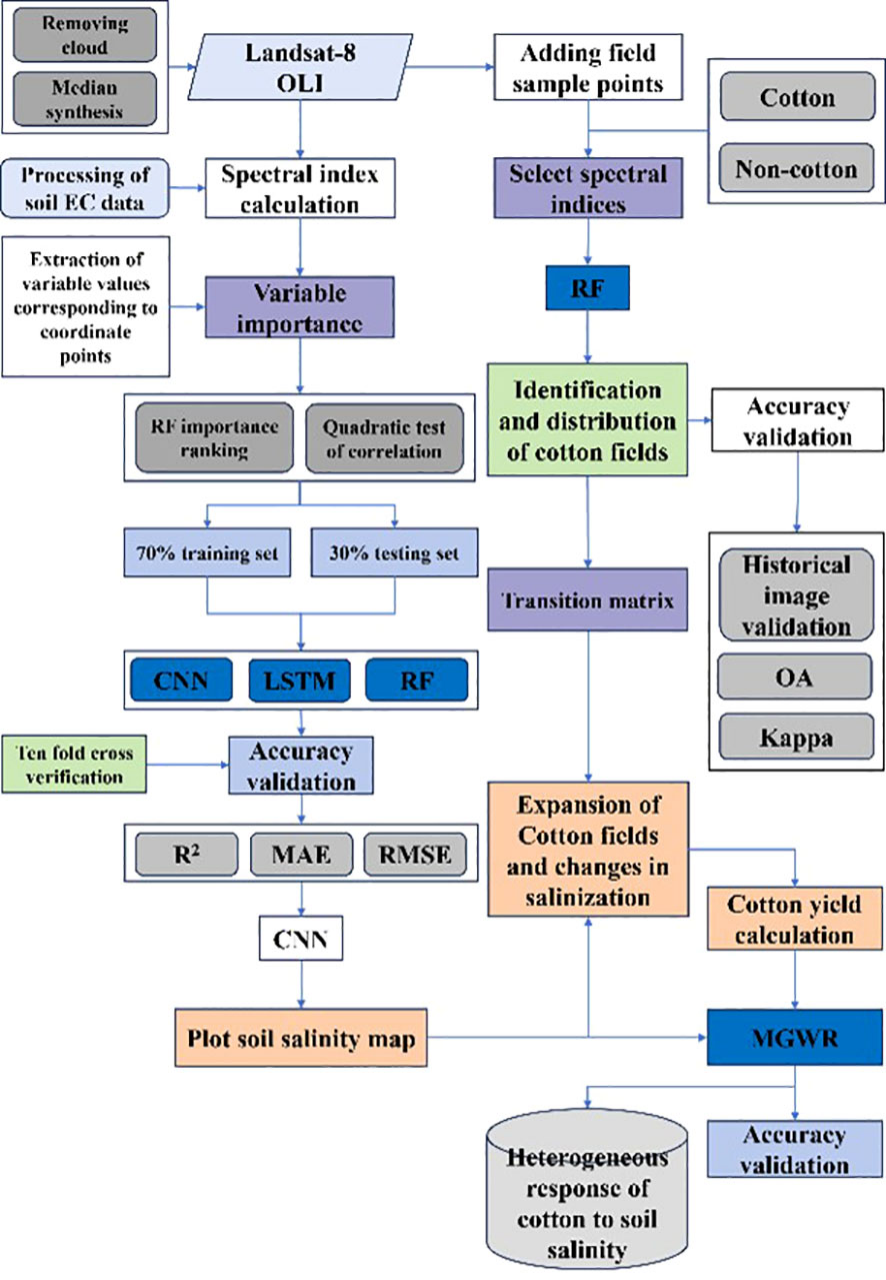
Research workflow diagram.

**Figure 1** presents the study's flowchart, which encompasses three principal sections: a) Construction of CNN, LSTM and RF inversion models for mapping of soil salinity distribution in 2013 and 2022; b) Identifying the distribution of cotton fields in 2013 and 2022, and calculating image-by-image metric yields of the cotton fields; and c) Analyzing the soil salinity status of the newly added cotton fields and exploring the spatial heterogeneous response relationship.

### 2.1 Study area

**Figure 2:**
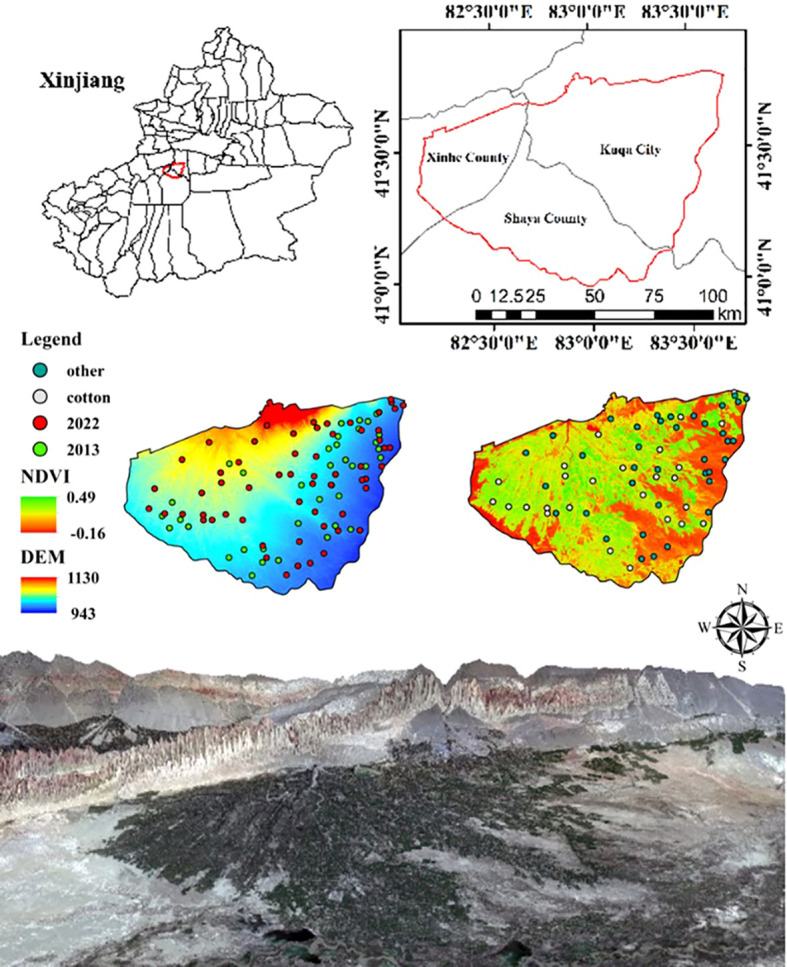
Schematic map of study area and distribution of sampling points.

The Wei-Ku Oasis is situated in the Aksu region of Xinjiang (81°28′30″~84°05′06″E、39°29′51″~42°38′01″N) (**Figure 2**). The oasis includes three counties: Xinhe, Shayar, and Kuqa. The salinization problem of soil in the Wei-Ku Oasis is prominent, with a total area of 523.76×10^4^ hm^2^ for the three counties. Shayar County has an average of 2667 hm^2^ of land planted with crops that do not yield profits annually. Additionally, land salinization has led to the degradation of grassland area, reaching 4.56×104 hm^2^. The degradation area of grassland in Xinhe and Kuqa counties has also reached 3.72×104 hm^2^ (Xueping, 2009). The climate of the Wei-Ku Oasis is characterized by a temperate continental arid climate, with an annual average evaporation of 1991.0 to 2864.3 mm and an annual average precipitation of only 51.3 mm. The multi-year average temperature ranges from 10.6°C to 14.8°C, with the highest and lowest temperatures recorded at 41.3°C and -28.7°C, respectively. High evapotranspiration ratios allow soil salts to accumulate.

### 2.2 Data sources

#### 2.2.1 Field soil sampling and laboratory analysis

The soil data sampling points were primarily selected based on field surveys, taking into account the topography, vegetation cover, and previous research results in the study area. This study selected 37 and 63 representative sampling points in the study area from August 25 to September 5, 2013, and from June 20 to July 10, 2022, respectively (with land use types recorded for the 2022 samples). Portable Global Positioning System (GPS) devices were used to record the geographical location of sampling sites. Soil samples were collected at each sampling point in a uniform manner within a 1-meter radius at a depth of 0-10 cm using a soil sampler. Five samples were collected and mixed together within this radius, and approximately 500 grams of the mixed sample were placed in labeled waterproof sealed bags. All soil samples were subjected to a series of treatments including natural drying, grinding, and sieving (2.0mm) in the laboratory. Twenty grams of processed soil and 100ml of distilled water were thoroughly shaken and left to stand for 24 hours. The electrical conductivity (EC) values of the leachate after standing were measured at 25°C using a multiple parameter measuring instrument (WTWinoLab® Multi3420 Set B, WTW GmbH, Germany).Following the removal of outliers, a total of 97 sample data points were obtained. Consequently, the EC value of the soil leachate can be employed as a reference index for the evaluation of soil salinity (Hardie and Doyle, 2012).

#### 2.2.2 Remote sensing data and processing

The Landsat-8 imagery used in the study came from Google Earth Engine (GEE).As the soil sampling periods are concentrated in late June, late August, and early September, we utilized cloud masking functions in GEE and selected Landsat-8 images with cloud cover below 40% from May to September to composite mean images for the years 2013 and 2022. The cotton field identification task involved synthesizing median composite images from mid-April to mid-November for the full growing season of cotton to obtain the 2013 and 2022 remote sensing images. Cotton yield prediction utilized median composite remote sensing images synthesized during the bolling period from July to mid-August.

### 2.3 Spectral bands selection for salinity modeling

Spectral reflectance characterizes the degree of soil salinization by the reflectance values at specific wavelengths; higher reflectance values indicate more severe soil salinization at the land surface (Al-Ali et al., 2021). Therefore, it is essential to include spectral reflectance in the model's input feature variables. We selected B1-B7 as feature variable input models from the raw spectral bands provided by the OLI sensor of Landsat-8.

#### 2.3.1 Vegetation indices

The spectral reflectance of vegetation under salt stress can serve as an indirect indicator of salt presence, especially in arid regions with low vegetation coverage, where its sensitivity is higher. Previous studies (Du et al., 2021) have indicated that vegetation indices are employed in machine learning methods to effectively elucidate the close relationship between vegetation and soil salinity, thereby enhancing the accuracy of soil salinity information retrieval. The formula for calculating the vegetation index selected for this study is shown in **Table 1**.

**Table 1:** Vegetation indices.

Vegetation Index	Formula	Reference
NDVI	(NIR−R)/(NIR+R)	(Hong et al., 2023)
EVI	$2.5\left[\frac{\left(NIR-R\right)}{\left(NIR+6\times R-7.5\times B+1\right)}\right]$	(Li et al., 2023)
DVI	NIR−R	(Haq et al., 2023)
GDVI	$\left(NIR^{2}-R^{2}\right)/\left(NIR^{2}+R^{2}\right)$	(Zhang et al., 2023)
MSAVI	$\left[2NIR+1-{\left({\left(2NIR+1\right)}^{2}-8\left(NIR-R\right)\right)}^{0.5}\right]/2$	(Guo et al., 2023)
NLI	$\left(NIR^{2}-R\right)/\left(NIR^{2}+R\right)$	(Huete et al., 2002)
RVI	NIR/R	(Abulaiti et al., 2022)
OSAVI	(NIR−R)/(NIR+R+0.16)	(Tian and Min, 1998)
ENDVI	(NIR+SWIR1−R)/(NIR+SWIR2+R)	(Song and Park, 2020)
IPVI	NIR/(NIR+R)	(Moradi et al., 2022)
GARI	{NIR−[G+γ×(B−R)]}/{NIR+[G+γ×(B−R)]}	(Gitelson et al., 1996)
GVMI	[(NIR+0.1)−(SWIR1+0.02)]/[(NIR+0.1)+(SWIR1+0.02)]	(Xiao et al., 2023)

B represents the blue band; G represents the green band; R represents the red band; NIR represents the near-infrared band; SWIR1 represents the shortwave infrared band 1; SWIR2 represents the shortwave infrared band 2; γ is the parameter controlling atmospheric correction.

#### 2.3.2 Soil indices

Soil texture is one of the fundamental conditions for salt accumulation. Gypseous desert soil is a type of desert soil with a distinct layer enriched in gypsum, widely distributed in the Xinjiang region. In addition to its elevated gypsum content, the substance in question also exhibits a high salt content. High soluble salt content in the soil is reflected below the gypsum accumulation layer, typically forming sulfate salts at the gypsum surface. Gypsum index (GYEX) can reflect the salt content status in bare soil areas. Carbonate index (CAEX) and clay index (CLEX) reflect the soil's water retention capacity based on the content of carbonate and clay in the soil, thus indicating the salt migration capability. The formulae used in this study to calculate the soil indices are shown in **Table 2**.

**Table 2:** Soil indices.

Soil index	Formula	Reference
CLEX	SWIR1/SWIR2	(Prout et al., 2021)
GYEX	(SWIR1−NIR)/(NIR+SWIR2)	(Taghizadeh-Mehrjardi et al., 2014)
CAEX	G/B	(Taghizadeh-Mehrjardi et al., 2014)
BI	${\left(G^{2}+B^{2}\right)}^{0.5}$	(Rafik et al., 2022)

#### 2.3.3 Salinity indices

Salinity indices can directly reflect the degree of soil salinization either on spectral bands or by influencing canopy reflectance. The interaction among soil salinity, water content, and vegetation maintains the dynamic equilibrium of ecosystems. In this study, a total of 20 salt indices proposed by various researchers were calculated, and the formulas are shown in **Table 3**.

**Table 3:** Salinity indices.

Salinity index	Formula	Reference
S1	B/R	(Allbed et al., 2014)
S2	(B−R)/(B+R)	(Allbed et al., 2014)
S3	G×R/B	(Allbed et al., 2014)
S5	B×R/G	(Allbed et al., 2014)
S6	R×NIR/G	(Allbed et al., 2014)
S7	(SWIR1−SWIR2)/((SWIR1+SWIR2)	(Bannari et al., 2008)
S8	(G+R)/2	(Nicolas and Walter, 2006)
S9	(G+R+NIR)/2	(Nicolas and Walter, 2006)
SI	${\left(G+R\right)}^{0.5}$	(Khan et al., 2005)
SI1	${\left(G\times R\right)}^{0.5}$	(Khan et al., 2005)
SI2	${\left(G^{2}+R^{2}+NIR^{2}\right)}^{0.5}$	(Khan et al., 2005)
SI3	${\left(G^{2}+R^{2}\right)}^{0.5}$	(Khan et al., 2005)
SI4	SWIR1/NIR	(Khan et al., 2005)
SIT	$\left(\frac{R}{NIR}\right)\times 100$	(Jia et al., 2022)
SSSI1	R−NIR	(Bannari et al., 2008)
SSSI2	(R×NIR−NIR×NIR)/R	(Bannari et al., 2008)
NDSI	(NIR−SWIR1)/(NIR+SWIR1)	(Ghazali et al., 2022)
CRSI	${\left[\frac{NIR\times R-G\times B}{NIR\times R+G\times B}\right]}^{0.5}$	(Ma et al., 2023)

#### 2.3.4 Other indices

Considering that NDBI and MNDWI can characterize the wetness or dryness of water content, we added NDBI and MNDWI indices as covariates, with the calculation formulas shown in **Table 4**.

**Table 4:** NDBI and MNDWI.

Other index	Formula	Reference
NDBI	(SWIR1−NIR)/(SWIR1+NIR)	(Guo et al., 2015)
MNDWI	(B−SWIR1)/(B+SWIR1)	(Moisa et al., 2022)

### 2.4 Model construction

#### 2.4.1 Convolutional neural network

**Figure 3:**
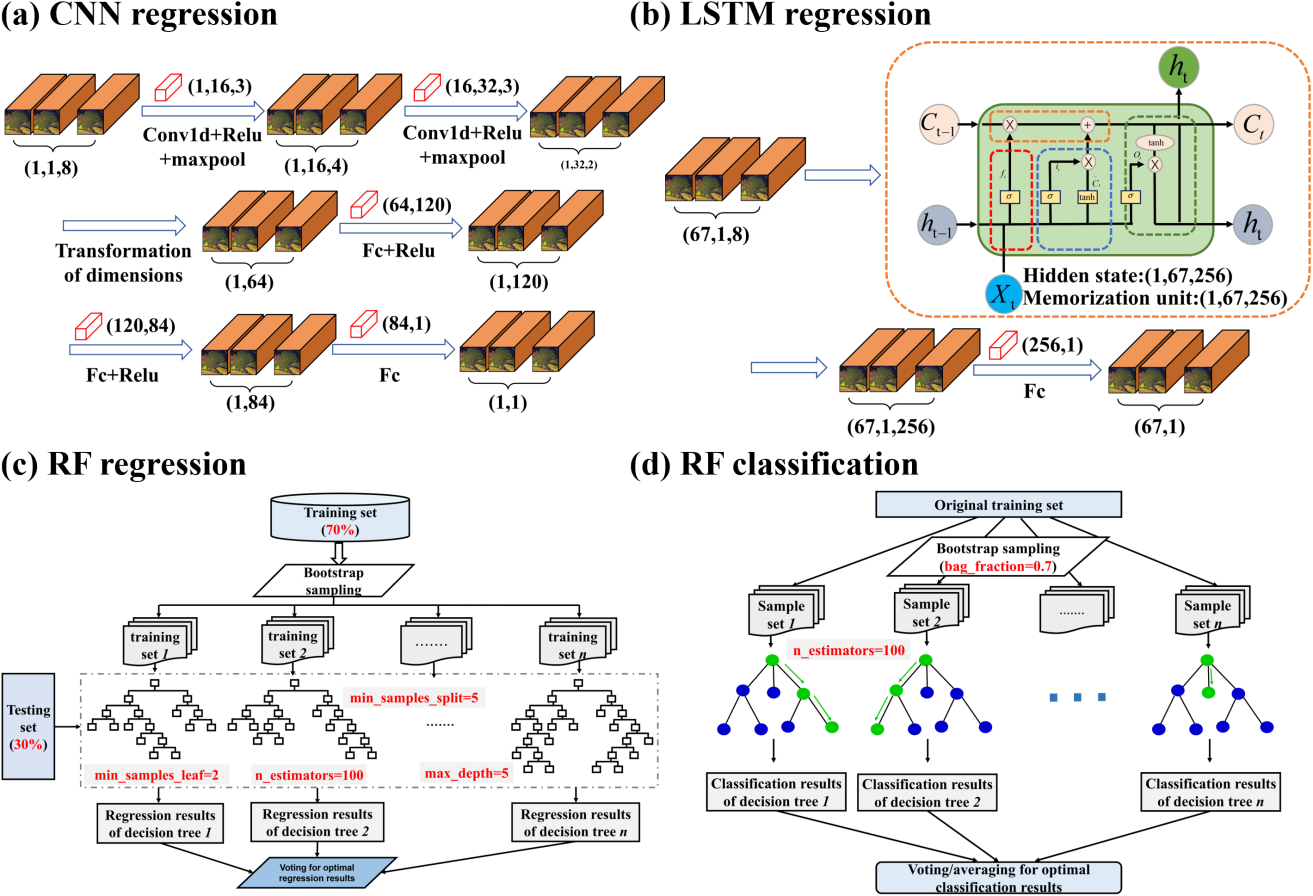
Schematic diagram of the model used in this paper Figures **(A–C)** respectively show the structures of the models used for soil salinity prediction, while figure **(D)** shows the structure of the Random Forest model used for crop classification.).

CNN (LeCun et al., 1998) is a type of artificial neural network widely used in image recognition (**Figure 3**). The core features of CNN include their design with local connections and weight sharing, which reduce the number of network parameters and alleviate overfitting issues. Considering the one-dimensional nature of our measured soil salinity data, we constructed a one-dimensional convolutional neural network to address the regression prediction problem of soil salinity. This model can effectively extract local pattern features from time series data and achieve good convergence within a relatively short time. The model consists of two one-dimensional convolutional layers. The first layer uses 16 convolutional kernels with a kernel size of 3, a stride of 1, and padding of 1. The second layer uses 32 convolutional kernels with a kernel size of 3, a stride of 1, and padding of 1. Following the convolutional layers is a one-dimensional max-pooling layer with a pooling window size of 2, which reduces the size of the feature maps. After the convolutional and pooling operations, the feature maps are flattened and passed to two fully connected layers, with the first layer having 120 nodes, the second layer having 84 nodes, and the final output being a regression value. The model employs the ReLU activation function to introduce non-linearity, uses the Adam optimizer with a learning rate of 0.0006, and has a loss function of mean squared error (MSE), with training conducted over 300 epochs.

#### 2.4.2 Long short-term memory

LSTM (Hochreiter and Schmidhuber, 1997) is a specific type of recurrent neural network (RNN) (**Figure 3**). Unlike standard RNN, LSTM has memory units capable of learning long-term dependencies and can effectively handle sequential data. The core component of the model is the LSTM layer, which has an input feature dimension of 8 and maps to 256 hidden units. The LSTM layer is responsible for capturing both short-term and long-term dependencies in the time series. Next, a fully connected layer maps the output of the LSTM to the regression result. During the forward propagation process, the LSTM’s hidden states and memory cells are initialized at each iteration, with the final step involving the generation of the final prediction result through the fully connected layer. The model uses mean squared error (MSE) as the loss function, Adam as the optimizer, with a learning rate set to 0.0009, and is trained over 5000 epochs.

#### 2.4.3 Random forest

RF (Breiman, 2001) is mainly used for classification and regression problems (**Figure 3**). The core idea of Random Forest includes random sampling, random feature selection, and prediction through majority voting. The parameters of the Random Forest model were set to 100 decision trees, a random seed of 5, a maximum depth of 5, a minimum of 5 samples required to split a node, and a minimum of 2 samples required to be at a leaf node. The model was then trained.

### 2.5 Accuracy validation of soil salinization inversion

In this study 70% samples are used as training set and 30% samples are used as test set. The optimal parameters for all models were obtained by tenfold cross-validation. The performance of the model is assessed by the coefficient of determination ( $R^{2}$ ), mean absolute error ( MAE ), and root mean square error ( RMSE ), with the respective formulas as follows:


(1) $$\begin{array}{c} R^{2}=1-\frac{\sum_{i}{\left({\hat{y}}_{i}-y_{i}\right)}^{2}}{\sum_{i}{\left({\bar{y}}_{i}-y_{i}\right)}^{2}} \end{array}$$

(2) $$\begin{array}{c} MAE=\frac{1}{m}\sum_{i=1}^{m}|{\hat{y}}_{i}-y_{i}| \end{array}$$

(3) $$\begin{array}{c} RMSE=\sqrt{\frac{1}{m}\sum_{i=1}^{m}{\left(y_{i}-{\hat{y}}_{i}\right)}^{2}} \end{array}$$

In the formulas: $y_{i}$ represents the measured soil EC value; ${\hat{y}}_{i}$ denotes the predicted soil EC value; ${\bar{y}}_{i}$ stands for the mean measured soil EC value; and m represents the number of sampling points.

### 2.6 Cotton planting distribution and yield prediction

#### 2.6.1 Environmental covariates

When extracting cotton planting areas, we selected Landsat-8 bands B1-B7, as well as NDVI and EVI spectral indices to identify cotton planting regions (Wang et al., 2017). The calculation formulae for NDVI and EVI are shown in **Table 1**.

#### 2.6.2 Construction of classification models

Selecting the Random Forest model for cotton classification on the GEE platform, as described previously. We set the specific parameters of the Random Forest model in the GEE as follows: the number of trees in the Random Forest is set to 100, with a sampling proportion of 0.7 for the cotton classification task (**Figure 3**). Similarly, we divided the sample 70:30.

#### 2.6.3 Accuracy validation of cotton field classification

To evaluate the classification performance of cotton fields, the confusion matrix is used in this study. Currently, the confusion matrix represents the most widely utilized methodology for evaluating the accuracy of classification algorithms. This method includes four evaluation metrics: producer's accuracy, user's accuracy, overall accuracy, and Kappa coefficient. The calculation formulas are as follows:


(4) $$\begin{array}{c} OA=\frac{TP+TN}{TP+TN+FP+FN} \end{array}$$

(5) $$\begin{array}{c} Kappa=\frac{PA-PE}{1-PE} \end{array}$$

(6) $$\begin{array}{c} UA=\frac{TP}{TP+FP} \end{array}$$

(7) $$\begin{array}{c} PA=\frac{TP}{TP+FN} \end{array}$$

In these formulas, TP , TN , FP , and FN represent the counts of true positives, true negatives, false positives, and false negatives, respectively; PA is the accuracy of the model on the actual data, PE is the expected accuracy under random classification.

#### 2.6.4 Cotton yield prediction

High correlation between cotton yield and NDVI, which has been extensively validated in practice (Liu et al., 2015; Meng et al., 2019). Therefore, in this study, raster image estimation is used to expand county-level yield maps to pixel-level spatial distributions. By combining data from statistical yearbooks and government bulletins on cotton economic yield, the average cotton yield at the pixel level for three county-level divisions in the study area is obtained (Cai and Sharma, 2010) (the average yield of Xinhe County and Shayar County is used to replace the missing cotton yield per mu in Kuqa City in 2022). Then, the cotton yield of individual pixels is expanded using the NDVI index of cotton. The calculation formula is as follows:


(8) $$\begin{array}{c} Y_{P}=Y_{avg}\times \frac{NDVI_{P}}{NDVI_{avg}} \end{array}$$

Where $Y_{P}$ represents the cotton yield of a single pixel (kg·hm^-2^); $Y_{avg}$ is the average cotton yield at the county level, obtained from statistical yearbooks and government bulletins; $NDVI_{P}$ is the NDVI value during the flowering period of cotton; $NDVI_{avg}$ is the average NDVI value during the flowering period at the county level.

### 2.7 MGWR model

#### 2.7.1 Model principle

The MGWR model is a geographically weighted regression model used to explore spatial heterogeneity in geospatial data (Fotheringham et al., 2017). In contrast to traditional global regression models, the MGWR model is capable of capturing local spatial correlations in spatial data, thereby enhancing the model's explanatory power and prediction accuracy. This model allows regression coefficients to vary with spatial location, thus better understanding the nonlinear relationships and spatial heterogeneity in spatial data.


(9) $$\begin{array}{c} y_{i}=\beta_{0}\left(u_{i},v_{i}\right)+\sum_{j=1}^{p}\beta_{j}\left(u_{i},v_{i}\right)x_{ij}+\epsilon_{i} \end{array}$$

Where $\beta_{0}\left(u_{i},v_{i}\right)$ and $\beta_{j}\left(u_{i},v_{i}\right)$ represent the locally varying regression coefficients, and $\epsilon_{i}$ denotes the error term. Typically, $\beta_{0}\left(u_{i},v_{i}\right)$ and $\beta_{j}\left(u_{i},v_{i}\right)$ can be weighted averaged using an appropriate spatial weighting function to reflect the spatial heterogeneity of spatial data.

#### 2.7.2 Accuracy validation of MGWR model

We use the adjusted coefficient of determination $R_{adjusted}^{2}$ to validate the rationality of the MGWR model. The formula for adjusted $R_{adjusted}^{2}$ is as follows:


(10) $$\begin{array}{c} R_{adjusted}^{2}=1-\frac{\left(1-R^{2}\right)\left(n-1\right)}{n-p-1} \end{array}$$

Where: $R_{adjusted}^{2}$ is the adjusted coefficient of determination; n is the sample size; p is the number of independent variables used in the model.

## 3 Results

### 3.1 Digital mapping of soil salinization inversion

#### 3.1.1 Descriptive statistics of soil salinity

The EC in the study area exhibits a considerable range, spanning from 0.17 to 117.9 dS/m, with a mean of 18.3 dS/m (**Table 5**). It is generally considered that a coefficient of variation (CV) less than 10% indicates weak variability, values between 10% and 100% represent moderate variability, and values greater than 100% indicate strong variability. The coefficient of variation is greater than 100%, indicating a high spatial variability of soil salinity (Hu et al., 2023). The pH value ranges from 6.463 to 8.644, with relatively low variability.

**Table 5:** Descriptive statistics of soil salinity.

Soil properties	Minimum	Maximum	Mean	Standard deviation	Variable Coefficient%
EC/(ds·m^-1^)	0.17	117.9	18.3	24.71	135%
pH value	6.463	8.644	7.582	0.364	4.8%

#### 3.1.2 Importance screening of soil salinity and environmental variables

**Figure 4:**
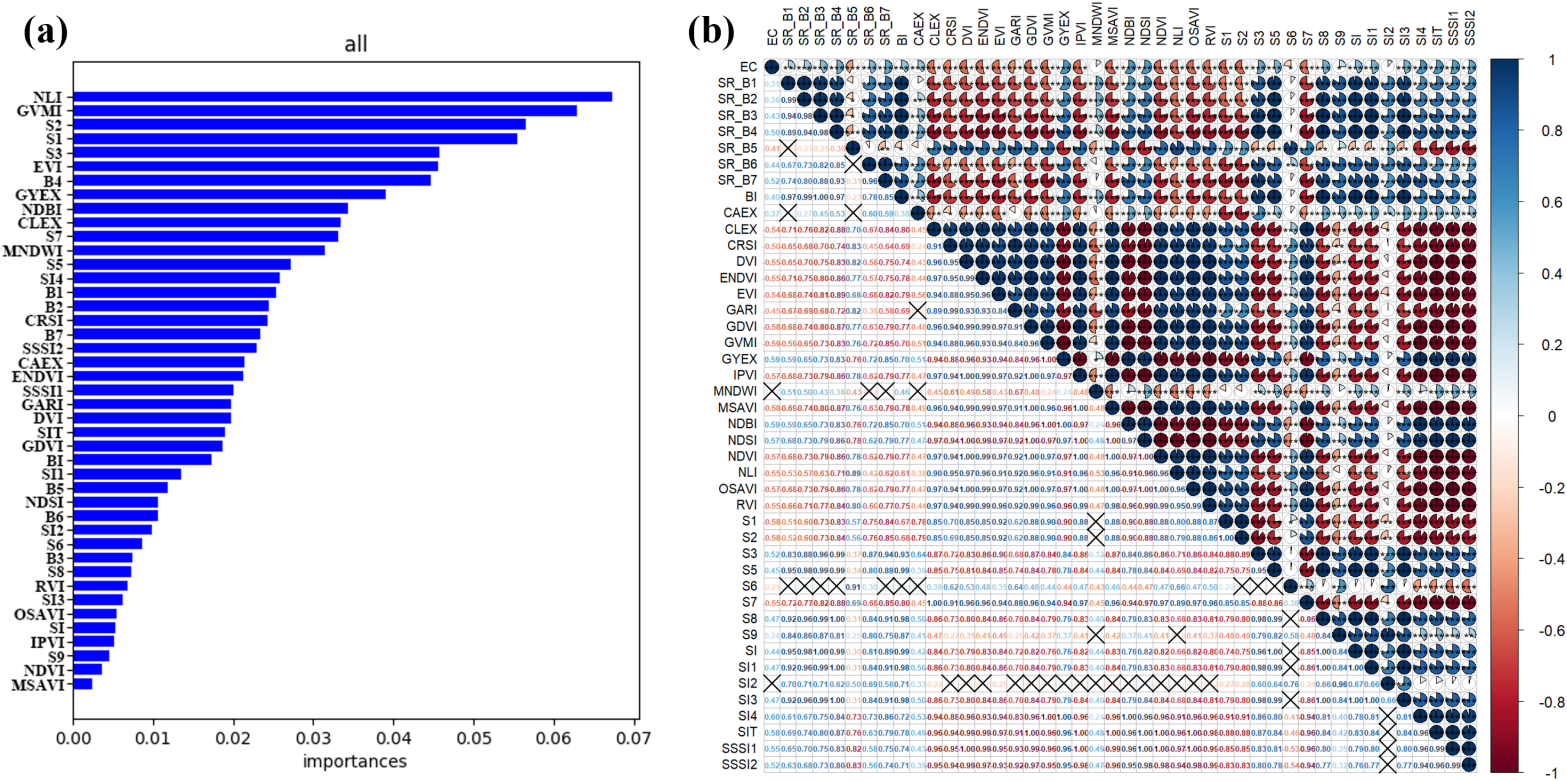
Importance and correlation of feature variables Figure **(A)** represents the feature importance selection used for model construction, while Figure **(B)** shows the Pearson correlation analysis of all feature variables. This analysis is used to further validate the selected features by checking if their correlation with soil salinity exceeds 0.5.).

We obtained the importance ranking of environmental variables by iterating 100 times using the random forest model (**Figure 4**), and selected the top eight important environmental variables (NLI, GVMI, S2, S1, S3, EVI, B4, GYEX) for modeling. This indicates that apart from salinity index, nonlinear vegetation index can also explain the EC content within the oasis, and soil index is also an important variable for predicting soil EC. Furthermore, the correlation coefficients between each variable and the soil EC value were obtained through Pearson correlation coefficient analysis. The Pearson correlation coefficients calculated using all feature variables were further validated, showing that the Pearson correlation coefficients for the eight feature variables used to construct the model were all above 0.5, indicating a strong correlation with soil salinity and improving the accuracy of the modeling.

#### 3.1.3 Comparison of soil salinity mapping and accuracy among different models

**Figure 5:**
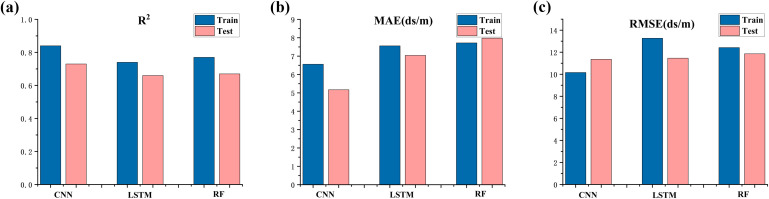
Comparison of model accuracy (**A** represents the model determination coefficient, **B** represents the mean absolute error, **C** represents the root mean square error, blue represents the accuracy verification of the soil salinity inversion model on the training set, and pink represents the accuracy verification on the test set).

Based on the selection of eight feature variables, CNN, LSTM, and RF models were established for soil salinization inversion. From the perspective of modeling accuracy of the three different models, all three models have achieved a good level of performance. RF is prone to limitations due to the step size when processing data, which may result in suboptimal performance in long-term prediction tasks and does not exhibit higher stability compared to deep learning models. Nevertheless, machine learning models represented by RF have a significantly lower training time cost compared to deep learning models, allowing for rapid predictions. LSTM aims to model temporal dependencies in sequential data, emphasizing global features. In contrast, CNN can capture subtle variations in data features, which allows it to better identify local features, resulting in superior performance in surface soil salinity prediction, with R^2^ values of 0.84 and 0.73 for the training and testing sets, respectively (**Figure 5**). Additionally, the MAE and RMSE values for the training set are 6.56 dS/m and 10.16 dS/m, respectively, and for the testing set, they are 5.17 dS/m and 11.36 dS/m, respectively.

**Figure 6:**
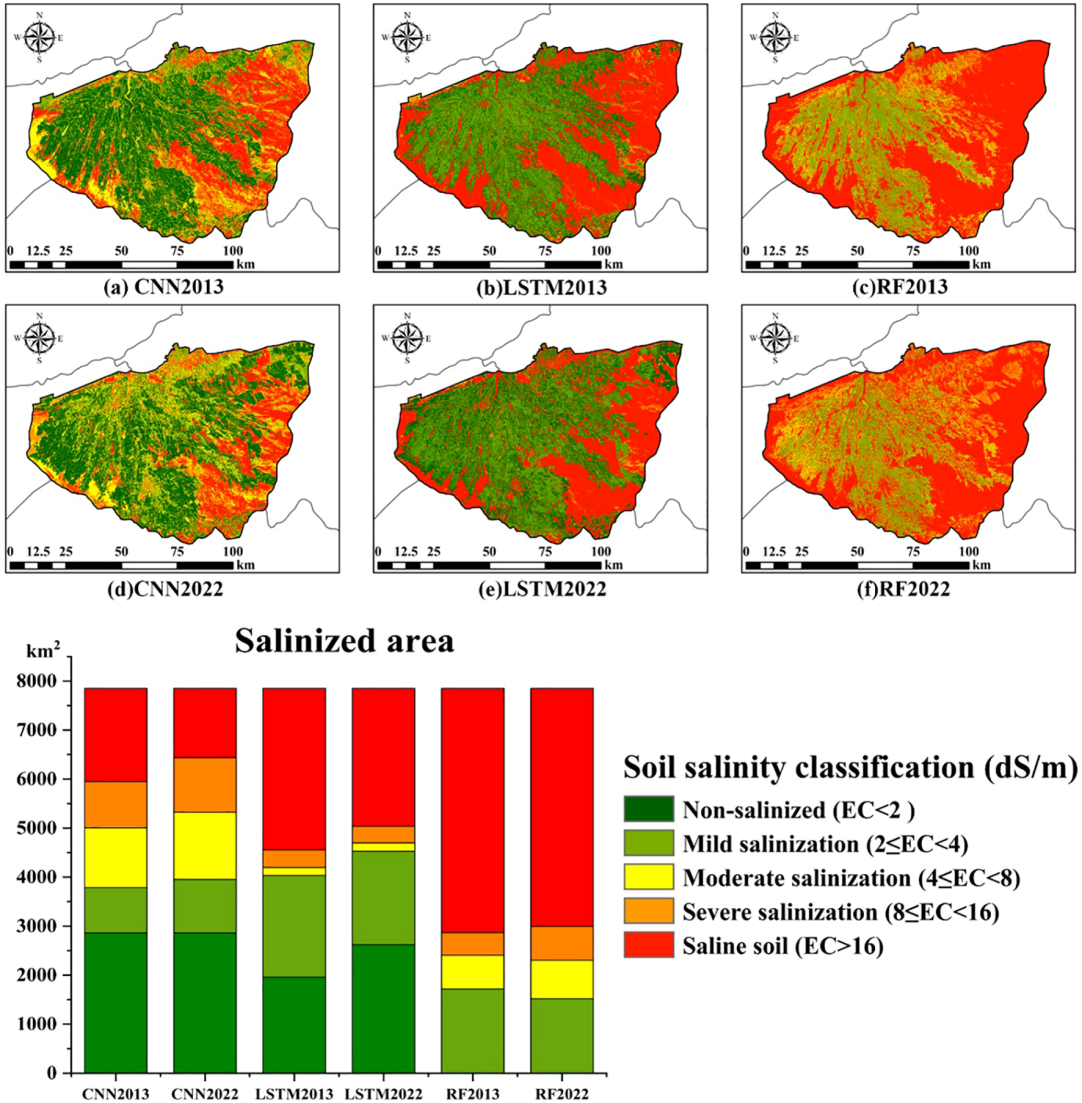
Soil salinity maps for 2013 and 2022 generated by different models, along with the proportions of different degrees of saline-alkali soil Figures **(A–C)** represent the soil salinity distribution maps for the year 2013, generated using the CNN, LSTM, and RF models, respectively. Figures **(D–F)** represent the soil salinity distribution maps for the year 2022, generated using the CNN, LSTM, and RF models, respectively.).

Soil salinity is classified into five categories based on the criteria for soil salinity levels: non-salinized (EC< 2), slight salinization (2< EC< 4), moderate salinization (4< EC< 8), severe salinization (8< EC< 16), and saline soil (EC > 16) (Scudiero et al., 2015). The three models previously described were applied to Landsat-8 images in order to obtain digital maps of soil EC for the Wei-Ku Oasis in 2013 and 2022. As shown in **Figure 6**, the spatial distribution characteristics derived from the three models are similar. Soils within the oasis have low salinity, while soils outside the oasis generally have high salinity. The RF model can only show a general trend in predicting soil salinity in the study area, which also demonstrates its tendency for instability in long-term prediction tasks. The LSTM model shows minimal local variation in predicting salinity within oases and deserts, which aligns with its focus on global features and neglects the depiction of local characteristics. In contrast, the CNN model can capture more spatial details and leverage its advantage in identifying local features of surface soil salinity, consistent with the results of Wang et al. (2023b), and effectively reveals the spatial heterogeneity of soil salinity in the study area.

According to CNN prediction results, non-salinized and slightly salinized areas are mainly concentrated within the oasis. The degree of salinization increases in the desert areas extending outward from the oasis edge’s desert interleaved belt. The deserts in the eastern part of the oasis are primarily moderately and severely salinized, while the central and western desert areas are mainly characterized by severe salinization and saline soil, consistent with the results of He et al. (2019). Analyzing over the time scale from 2013 to 2022, the saline-alkali area in the study region has decreased by 484.3 km² over the past decade. The areas of severe and moderate salinization have shown slight changes, while the area of mild salinization has increased by 164.59 km². The area of moderate salinization has not changed significantly because moderate salinization has decreased in desert areas, while some non-salinized and lightly salinized soils within the oasis have transitioned to moderate salinization. Overall, the soil salinization problem in the Wei-Ku Oasis has been alleviated and improved due to a series of government management measures.

### 3.2 Expansion of cotton fields and changes in soil EC values

#### 3.2.1 Cotton field identification

**Figure 7:**
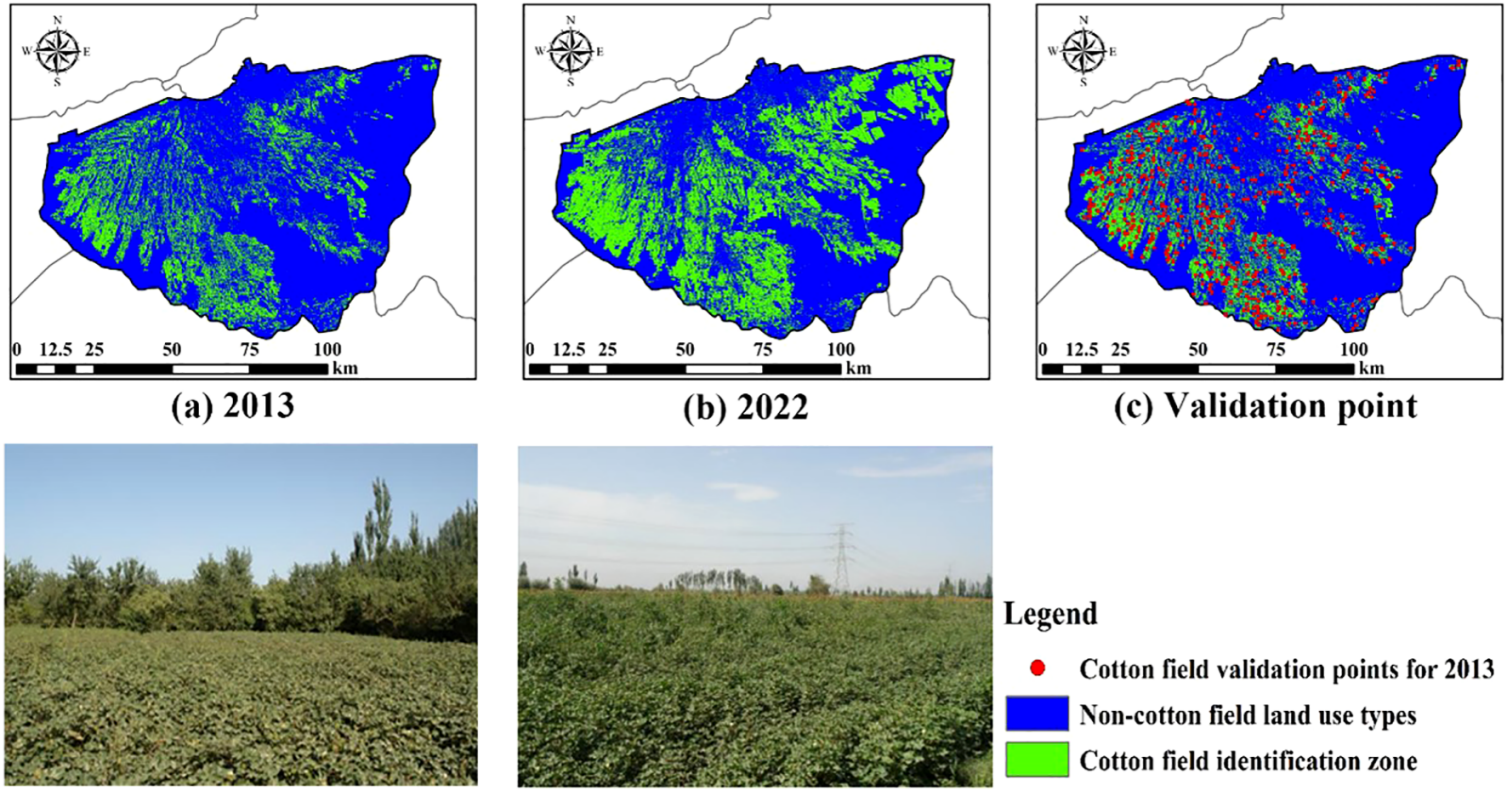
Cotton field identification and accuracy validation random points for 2013 and 2022 Figure **(A)** shows the distribution of cotton fields in 2013, figure **(B)** shows the distribution of cotton fields in 2022, and figure **(C)** shows the random validation points used for the cotton field distribution in 2013.).

The overall accuracy of the cotton field recognition model constructed using RF reached 94.4%, and the Kappa coefficient reached 88.88%. In order to ascertain the veracity of the model in 2013, 500 validation points were randomly generated within the classified results utilizing the ArcGIS 10.8 software (**Figure 7**). The validation points were imported into Google historical imagery for visual interpretation, and combined with historical on-site sampling images from the laboratory, achieving an accuracy of 90.6%, demonstrating good applicability.

#### 3.2.2 Changes in soil EC values in cotton fields

**Figure 8:**
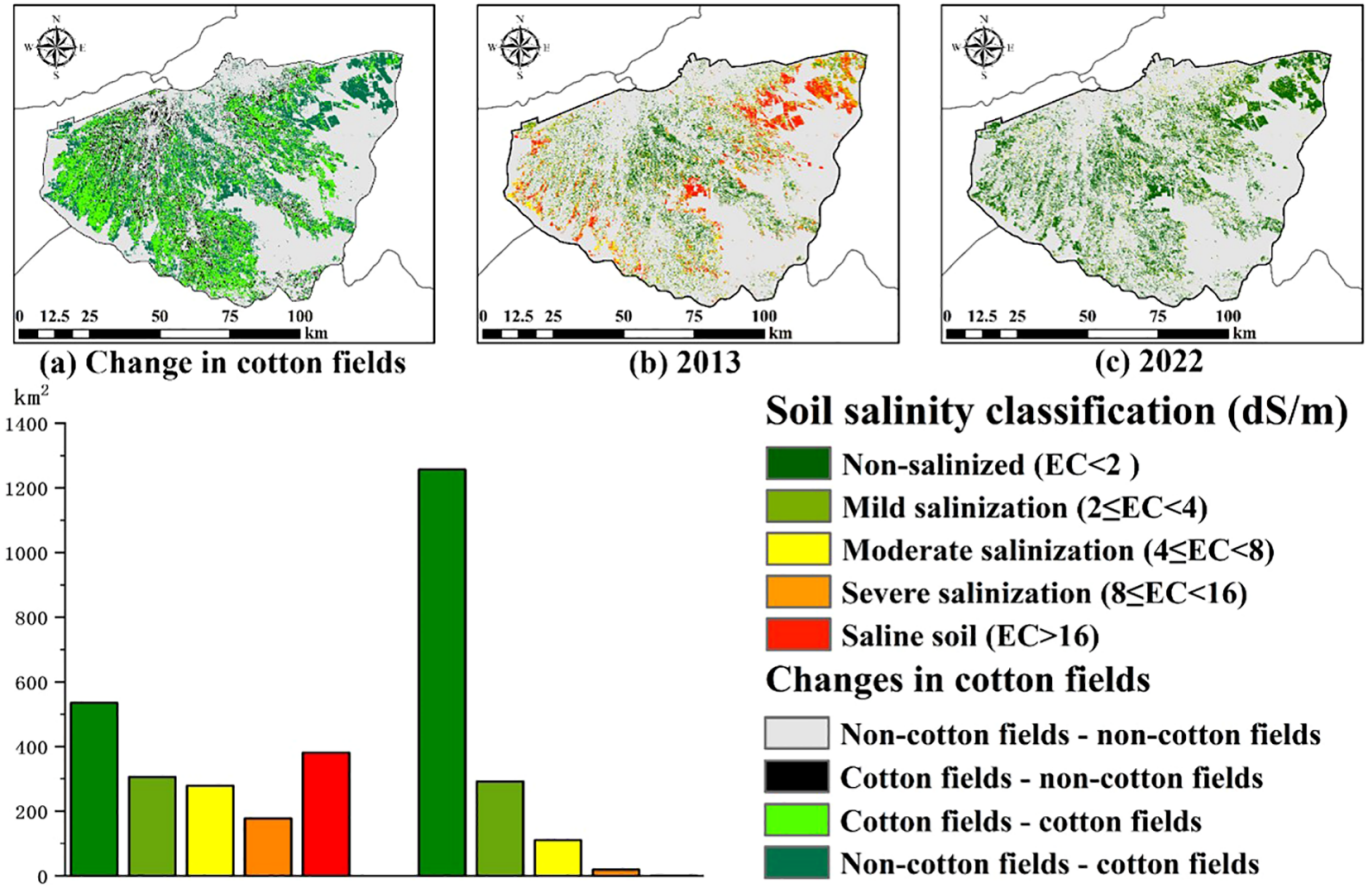
Cotton field expansion and improvement of saline-alkali soil area Figure **(A)** represents the distribution changes of cotton fields from 2013 to 2022, figure **(B)** shows the soil salinity levels in newly added cotton fields in 2013, and figure **(C)** shows the soil salinity levels in newly added cotton fields in 2022).

We calculated the changes in cotton field area using a land use transfer matrix (**Figure 8**). In 2022, 1334.68km² of cotton fields remained unchanged, 500.78km² of cotton fields converted to non-cotton fields, and another 1679.85km² of non-cotton fields were converted to cotton fields through changes in crops and cultivation of barren land. Among the areas converted to cotton fields, 1145.18km² of land had varying degrees of salinization in 2013, with severe salinization and saline-alkali areas reaching 177.91km² and 381.46km², respectively. However, by 2020, the non-salinized area had reached 1256.53km², with only 19.49km² and 1.12km² remaining for severe salinization and saline-alkali areas, respectively. This significant change demonstrates the positive role of cotton field expansion in addressing soil salinization issues.

### 3.3 Cotton field yield prediction and spatial heterogeneity response of soil salinity

**Figure 9:**
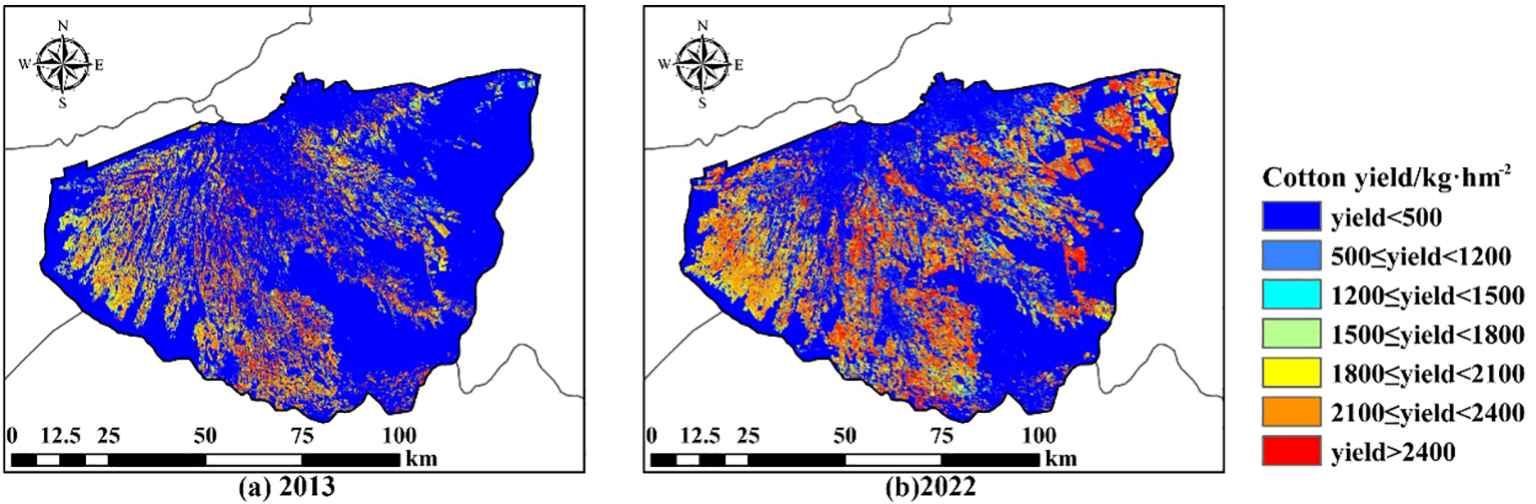
Cotton field yield (per pixel) in 2013 and 2022. **(A)** represents cotton field yield in 2013, and **(B)** represents cotton field yield in 2022.

First, the average cotton yield of three counties and NDVI index were used to plot the predicted cotton yield maps of the study area for 2013 and 2022 to demonstrate the growth status of cotton (**Figure 9**). In 2013, areas with low average cotton yields were mainly concentrated in the western and peripheral regions of the cotton fields. In 2022, areas with low average cotton yields were mainly concentrated in the western, southern, and newly reclaimed northeastern parts of the cotton fields.

**Figure 10:**
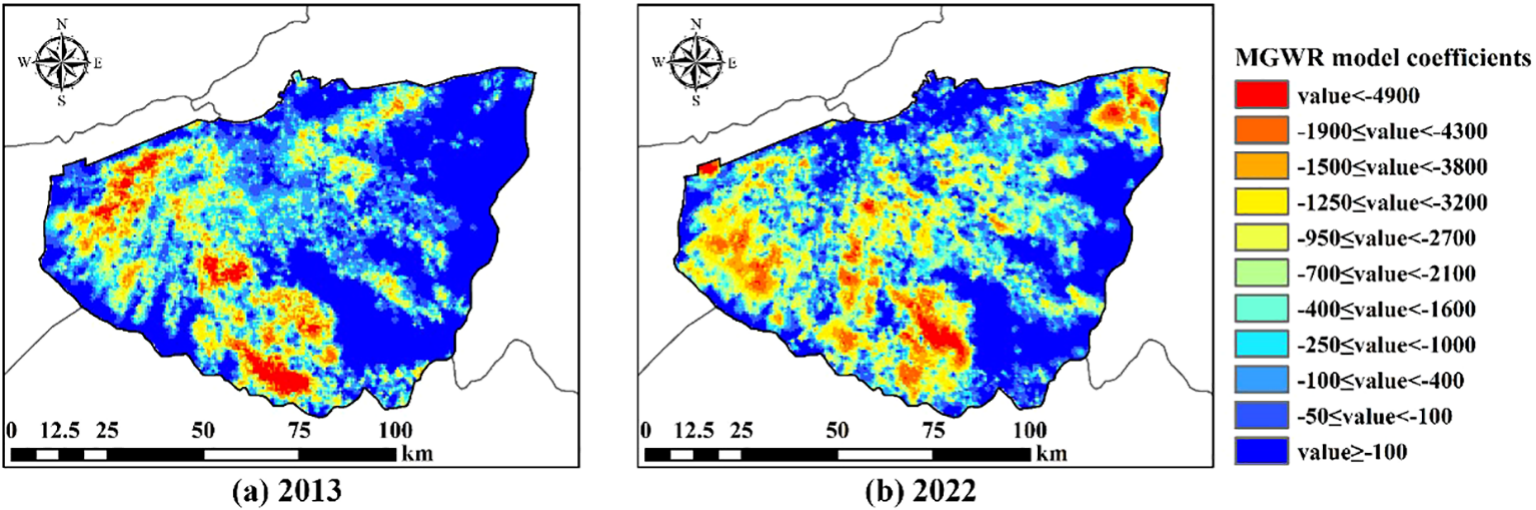
Spatial heterogeneity relationship between cotton field yield and soil salinity in 2013 and 2022. **(A)** represents cotton field yield and soil salinization spatial heterogeneity in 2013, and **(B)** represents cotton field yield and soil salinization spatial heterogeneity in 2022.

The predicted cotton yield map and soil salinity level map were resampled to 500m, and then the MGWR coefficients of soil EC value and cotton yield were calculated (**Figure 10**). According to the accuracy verification results, the R^2^_adjusted_ of the MGWR model in 2013 was 0.75, and in 2022 it was 0.86, reaching the expected level. Areas sensitive to soil salinity are mainly distributed in the western part of the Wei-Ku oasis, in Xinhe County and Shaya County, as well as in the northeastern part of Kuqa City, which has been newly reclaimed.

## 4 Discussion

### 4.1 Impact of environmental variables on soil salinity

Currently, a large number of research findings indicate that various environmental factors are highly effective in constructing soil salinity models. Zhu et al (2021) studied the relationship between vegetation indices and salinity, finding that soil salinity stresses crop growth, and vegetation greenness can indirectly reflect soil salinity levels. The highest contribution of vegetation index NLI in modelling soil salinity in arid zone was also found in the results of this study. Analysis of vegetation indices at a finer field scale (Polivova and Brook, 2021) showed that NDVI, EVI, GARI, and GDVI indices are highly sensitive to changes in vegetation. In this study, the EVI vegetation index also showed good sensitivity in the study area. The EVI index can eliminate the influence of soil and atmospheric aerosols, especially suitable for densely vegetated areas in oases, to obtain more accurate spectral information of vegetation canopy and assist in determining the soil salinity information under vegetation cover. A study conducted by Nguyen et al. (2021) in the Mekong Delta of Vietnam revealed that 13 indices, including EVI, CRSI, B5, B3, and B7, are the most effective in measuring soil salinity. The results of this study indicate that vegetation factors play an important role in improving model accuracy and monitoring soil salinity.

Salt index and soil index have been proven to be key variables in soil prediction in arid areas in previous studies. Wang et al. (2022a) pointed out in their another research that soil salinity directly changes the spectral reflectance in various bands. GVMI, GYEX, and DEM are among the factors with the highest contribution rates. The reason why DEM was not considered as a feature variable in this study is that the altitude fluctuation in the Wei-Ku oasis is not significant, and the differences are small, resulting in a low contribution rate to the prediction of soil salinity. The favorable performance of the salt index in this study corroborates the findings of Peng et al. (2019), namely, the salt index is the optimal and effective method for soil salinity monitoring in southern Xinjiang. Racetin et al. (2020) pointed out that salt indices S1 and S2 have the highest positive correlation, which can characterize the degree of salt influence in irrigated agricultural areas. In this study, S1 and S2, two salinity indices, also have relatively large contribution rates. S1 and S2 use the ratio of red band and blue band information, highlighting that in vegetation-covered areas, the red band has low absorption reflectance and a large ratio, while the opposite is true in desert areas.

The spectral characteristics of saline encrustations are correlated with soil roughness. The reflectance values increase with increasing soil salinity (Günal et al., 2021). The high reflectance characteristic of saline soils is readily discernible by satellite remote sensing, rendering it an invaluable tool for the remote monitoring of large-scale soil salinization. Gypsum desert soils are widely distributed in Xinjiang, with almost zero surface vegetation cover. Under intense wind erosion conditions, the gypsum layer often approaches or exposes the surface, making it prone to salt accumulation and the formation of salt crusts. Shortwave infrared produces strong reflections in these areas, resulting in relatively high gypsum index values. This suggests that the GVMI and GYEX indices are more effective at detecting soil salinity in arid areas with minimal vegetation cover or bare soil surfaces compared to other indices.

### 4.2 The impact of different models on soil electrical conductivity values

There is currently no universally acknowledged parameter solution for evaluating the effectiveness of machine learning (including deep learning) results, thus requiring a case-by-case analysis (Domingos, 2012). The performance of most algorithms on specific datasets highly depends on the learning parameters used to train them, yet parameter settings that yield optimal performance on one dataset may not generalize well to another (Bengio, 2012). From a single-model analysis perspective, LSTM is suitable for handling sequential data, capable of capturing long-term dependencies within the data, thereby aiding in better future value prediction. Hence, we made the initial attempt to predict soil salinity using LSTM in this study. Random forests, on the other hand, can handle a large number of features and exhibit a certain degree of robustness to missing values and outliers. They are effective in illustrating trends between low and high salinity areas, yet their ability to predict local soil EC values is not as prominent as CNN. Therefore, as a relatively mature deep learning model, CNN presents potential for exploring local information extraction across different study areas in future research, facilitating a better integration of soil salinity research with deep learning techniques.

### 4.3 Spatial heterogeneity response of soil salinization in cotton fields

The research findings indicate that the expansion of cotton fields can ameliorate soil salinization issues in the Wei-Ku oasis, particularly evident in reclaimed wasteland. To investigate the spatial heterogeneity of soil salinity and cotton field growth, the MGWR model was selected as the optimal method to capture these features.

The MGWR coefficients elucidate the spatial heterogeneity response of soil salinization to cotton growth, offering more detailed insights into the spatial relationship between the two factors. Based on the regression coefficients of soil EC values and cotton yield, we found a close correlation between soil EC values and cotton growth. Xinjiang has become an important cotton production base in China (Yang et al., 2020). For over two decades, cotton cultivation has advocated and employed plastic film-covered drip irrigation (Yang et al., 2022). It has been demonstrated that the duration of drip irrigation is a significant factor in the accumulation of soil salinity in cotton fields (Guan et al., 2019). In winter without irrigation, a large amount of salt concentrated in the subsoil will be redistributed upward, which leads to the accumulation of salt in the topsoil again in the spring (Qin et al., 2021), which is also the reason for the increase in salt in part of the inner oasis in result 3.1.3 (Zong et al., 2022). This in turn leads to reduced crop yields and reduced soil tillage capacity (Ajay, 2015). Consequently, cotton fields require substantial irrigation water. The prolonged percolation effects of long-term drip or surface irrigation cause soil salinity to migrate to the periphery of the oasis with the river flow. Thus, reducing irrigation water in the region may significantly increase soil salinization levels, leading to soil quality degradation, consistent with the findings of Yang et al (2022). Localized analysis reveals a widespread high negative correlation in Shaya County and the western part of Xinhe County. This area conforms to the aforementioned irrigation characteristics, with cotton yields relatively lower than in other regions. This suggests that changes in soil salinization levels are more likely to affect cotton growth in this area. The main reasons may be (1) the quality of irrigation water is partly from shallow groundwater, whose dissolved salt ions exacerbate the accumulation of salts. (2) The groundwater table is shallow and salt ions accumulate upwards through evaporation. (3) Drip irrigation not only fails to ensure sufficient irrigation water, but also keeps the soil in a relatively wet state, which is more conducive to the collection of salts in the upper layers. Therefore, a large amount of irrigation water is also needed to wash salt. Additionally, in newly cultivated cotton fields such as the northwest and northeast regions of the study area, the high sensitivity to soil EC values may be attributed to sparse surrounding vegetation cover, arid climate, and imperfect irrigation and salinity control measures, resulting in a strong response of cotton fields to soil salinity (Wang et al., 2023a). For cotton fields that are highly responsive to soil salinity, the following measures can be taken to reduce the impact of soil salinity on cotton growth. (1) Application of different types of organic, inorganic, and mixed amendments (Wang et al., 2022b). Organic amendments mainly include compost, humus, and plant residues (Scotti et al., 2015). They can increase soil organic matter (OM) content, thereby enhancing soil permeability (Chen et al., 2020). Additionally, they are rich in nitrogen and phosphorus, which can enhance the diversity of soil bacterial communities and strengthen interactions among microorganisms (Mao et al., 2022). Inorganic amendments mainly include gypsum, desulfurized gypsum, and aluminum sulfate, which are minerals or synthetically prepared compounds (Zhou et al., 2019). They alter the chemical properties of saline-alkaline soils through neutralization or ionic balance adjustments, thus improving the soil environment (Nan et al., 2016). Mixing organic and inorganic amendments can combine their advantages to achieve more effective results (Wang et al., 2024a). (2) Intercropping of cotton with halophytes. The main halophytes include Suaeda salsa and alfalfa (Medicago) (Díaz et al., 2018; Song and Wang, 2015). These plants can improve the physical and chemical properties of the soil by reducing bulk density, increasing soil porosity, and enhancing hydraulic conductivity (Ashraf et al., 2010),thereby promoting the leaching of soil salinity in saline-alkaline soils and reducing soil salt accumulation (Liang and Shi, 2021). (3) Adopt the Deep Vertical Rotary Tillage technique for cotton cultivation (Bai et al., 2024). Deep tillage creates a more favorable soil environment for root growth and development (Wang et al., 2020). It increases soil porosity, enhances the downward migration of salts, and limits the upward movement of water in soil capillaries, thereby reducing soil salinity and achieving the goal of increasing crop yields (Wei et al., 2020).(4) The impact of winter irrigation and water resource management (Li et al., 2020; Yang et al., 2022; Zhao et al., 2021). Winter irrigation effectively conserves soil moisture and inhibits salinity by pushing soil salts deeper into the soil layers, preventing salt return due to evaporation in the following year, thus providing favorable soil moisture and salinity conditions for crop growth (Xiao et al., 2018). Water resource management measures using subsurface drainage can reduce soil salinity and improve cotton emergence rates (Feng et al., 2019). Meanwhile, combining subsurface drainage with surface drainage can maximize the effectiveness of subsurface drainage. Therefore, improving the construction of drainage canals and enhancing water and fertilizer management (Wang et al., 2024b) can not only reduce the impact of soil salinization on cotton fields but also ameliorate soil salinization issues, improve soil quality and land productivity, and achieve scientific sustainable development.

### 4.4 Research limitations and future research directions

This study collected a total of 97 field samples; however, there is still a lack of soil salinization information validation in areas that are difficult to reach due to limited manpower (such as uninhabited areas or the interior of deserts). Additionally, due to the limited explanatory power of the model, these uninhabited areas still present some uncertainty. Furthermore, the model was constructed using primarily summer remote sensing imagery data, rendering it more suitable for mapping the spatial distribution of soil salinity for the same season across different years. Since the soil salt data we used were sampled in summer, further research will be conducted in different periods of the same year, and more measured samples will be collected to establish soil salinization inversion models respectively, and explore the spatial distribution characteristics of soil salt in different seasons of the year. Additionally, due to the limitations of the study period, higher-resolution satellite imagery, such as the 10-meter resolution Sentinel-1 and Sentinel-2 satellites and the 3-meter resolution Planet satellite, could not be utilized for soil salinity prediction and cotton field classification. Future research could consider the integration of multiple remote sensing images to enhance the accuracy of soil salinity prediction models and cotton field classification. To clarify the factors influencing cotton field yield, future research will consider the spatial heterogeneity of various influencing factors, including soil moisture content, precipitation, temperature, and groundwater level. Data-driven and inductive machine learning can capture information and extract patterns from the ever-growing geospatial data streams, demonstrating strong data adaptability; however, it lacks theoretical support and has weak interpretability. Future research could explore how to combine remote sensing physical process modeling with flexible data-driven modeling, aiming to develop a dual-driven quantitative remote sensing model that couples physical mechanisms with machine learning models, which may help solve the challenges of quantitative analysis of remote sensing data.

## 5 Conclusions

Based on the measured sample data and remote sensing image data, various deep learning and machine learning inversion models were constructed to map soil salinity in the Wei-Ku oasis, and the changes in soil EC values and spatial responses were investigated in relation to the expansion of cotton fields. The main results obtained are as follows:

(1) The CNN model showed higher accuracy as well as applicability, with an R^2^ of 0.84 for the training set and 0.73 for the test set. it can better mine the spatially localized information of soil salinity compared with other models.
(2) The expansion of cotton fields also significantly improved the soil salinity problem in the study area. The area of new cotton fields with heavy salinity and saline soils decreased from 177.91km^2^ and 381.46km^2^ to 19.49km^2^ and 1.12km^2^ from 2013 to 2022.The saline soil in the arid zone can be fully utilized by planting salt-tolerant crops.
(3) In 2013, the low yielding cotton fields were mainly located in the western part of the Wei-Ku oasis. In 2022, the low yielding areas of the cotton fields appeared in the southern and northeastern part of the newly reclaimed cotton fields in addition to the western part of the study area.
(4) The areas of cotton fields that are more sensitive to salinity alteration were explored by the MGWR model. The highly negatively correlated areas are located in the western and northeastern newly reclaimed areas of the study area, which provides a more scientific theoretical basis for the zonal management of salinization and promotes the sustainable development of oasis ecology and agriculture.

## Data Availability

The original contributions presented in the study are included in the article/supplementary material. Further inquiries can be directed to the corresponding author.
